# Two-stage transformer encoder and attention-guided GRU-based residual correction for short-term load forecasting

**DOI:** 10.3389/frai.2026.1935983

**Published:** 2026-10-19

**Authors:** Radhika Chandrasekaran, Senthil Kumar Paramasivan

**Affiliations:** School of Computer Science Engineering and Information Systems, Vellore Institute of Technology, Vellore, Tamil Nadu, India

**Keywords:** attention mechanism, encoder, gated recurrent unit, residual learning, short-term load forecasting, transformer

## Abstract

Accurate short-term load forecasting (STLF) plays a vital role in reliable power system planning and operations. The increasing electricity demand is driven by electrification, digitalization, population, and economic growth, which necessitates the need for accurate short-term load forecasting to ensure the real-time balance between supply and demand. Traditional forecasting approaches struggle to capture long-range temporal dependencies, and correcting the forecasting errors remains challenging due to increasing demand and dynamic load behavior. To address this limitation, this study proposes a two-stage forecasting architecture (transformer encoder and attention-guided GRU-based residual corrector [TE-AG-GRU-RC]) for short-term load forecasting. In the first stage, a transformer encoder (TE) processes historical load and exogenous features to generate the base forecast and capture long-range temporal dependencies from historical load sequences. In the second stage, an attention-guided GRU residual correction mechanism (AG-GRU-RC) captures short-term dynamics and refines the initial prediction with the temporal information derived from the transformer encoder. The final forecast is attained by combining the predicted residual with the base forecast, enabling the first stage to model dominant load patterns, while the second stage corrects the remaining errors. The proposed framework is evaluated using real-world multivariate hourly time series datasets from two geographically distinct regions. The forecasting performance of the model is evaluated against several baseline models, such as recurrent neural network (RNN), long short-term memory (LSTM), Gated Recurrent Unit (GRU), temporal fusion transformer (TFT), and ResidualLSTMPlus, to demonstrate its effectiveness. The proposed model attains mean absolute error (MAE), mean squared error (MSE), root mean squared error (RMSE), mean absolute percentage error (MAPE), and coefficient of determination (*R*^2^) values of 0.0297, 0.0014, 0.0376, 3.58, and 0.879, respectively, on the Tamil Nadu test data, and 0.0334, 0.0023, 0.0482, 4.99, and 0.790, respectively, on the Panama test data. These findings demonstrate the generalizability of the proposed model across both datasets with low prediction error and improved forecasting accuracy.

## 1 Introduction

The global energy demand is rising rapidly due to advancements in technology and expansion of metropolitan regions. Electricity is necessary for all aspects of our everyday life, and accurate short-term load forecasting is essential for reliable energy management, economic scheduling, power system planning, and operations. Moreover, the intermittent nature of renewable energy sources indicates the necessity of short-term load forecasting to predict energy demand fluctuations effectively (Mathumitha et al., 2024). The power utilization efficiency should be guaranteed by the integration of distributed renewable energy sources, providing intelligent demand-side management (Rao et al., 2024). Several external factors, such as weather and the introduction of new energy consumption scenarios, can influence electricity demand, producing load fluctuations that lead to non-linearity, which may increase the complexity of short-term load forecasting (STLF) (Dong et al., 2025). Accurate forecasting can avoid blackouts and brownouts to ensure a stable power supply. It allows utilities to optimize the power plant dispatch, avoiding unnecessary starts and stops of generators that might save fuel, reduce carbon emissions, and minimize operational costs. Demand-side management enables utilities to meet reliable peak demand by optimizing costs and renewable integration by flattening the load curve and promoting energy conservation.

Traditional statistical, machine learning, and deep learning approaches have distinct advantages and limitations. In short-term load forecasting, traditional models, such as autoregressive integrated moving average (ARIMA) (Fard and Akbari-Zadeh, 2014), seasonal autoregressive integrated moving average (SARIMA) (Anh et al., 2024), and exponential smoothing (Christiaanse, 1971), are mostly used owing to their strong statistical behavior and simplicity. The assumptions of linearity and stationarity in traditional statistical models are major challenges in handling electricity demand data. Additionally, the complexity in handling large datasets and limited adaptability to dynamic environments need to be addressed in traditional statistical methods. To address these challenges, machine learning approaches have emerged to capture non-linear relationships and complex interactions among the influencing factors when dealing with dynamic load patterns and high-dimensional data. Conventional machine learning approaches, such as artificial neural network (ANN) (Azadeh et al., 2014), Random Forest (Xuan et al., 2021), Support Vector Regression (Chen et al., 2017), and LightGBM-XGBoost (Yao et al., 2022), are commonly applied in short-term load forecasting. However, the challenges with machine learning approaches are that they cause overfitting, high data dependency, limited interpretability, struggle to capture complex temporal dynamics, and sensitivity to hyperparameter tuning. The transition toward renewable energy sources necessitates the need for deep learning approaches in short-term load forecasting, as traditional approaches struggle to capture non-linearities in the dynamic load. Recurrent models, such as recurrent neural network (RNN) (Veeramsetty et al., 2022), LSTM (Masood et al., 2024), and GRU (He et al., 2025), can automatically extract complex patterns and temporal dependencies from large datasets. Advanced models, such as the Informer transformer, are efficient for long-sequence time series forecasting (Zhou et al., 2021). The temporal fusion transformer (TFT) focuses on multi-horizon forecasting and emphasizes interpretability (Lim et al., 2021). The major challenge with deep learning recurrent networks is vanishing gradients, which limit the model’s ability to learn long-term temporal dependencies and capture global dependencies across long time horizons. It also requires additional training and interpretability.

Conventional single-model approaches have limited capability in capturing non-stationarity, multi-scale, and volatility. Hence, the necessity of integrating models has increased to benefit from the strengths of different models through the integration of multiple learning paradigms to improve predictive performance. It also penalizes significant model complexity and training overhead. The hybrid models include the maximum mutual information coefficient (MIC), long short-term memory (LSTM) with improved temporal convolutional network (TCN) (Mu et al., 2025), Bayesian-optimized Bi-LSTM with multi-head attention mechanism (Jiang and Xie, 2026), XGBoost-ANN (Harikrishnan and Sreedharan, 2025), stacked LSTM (Vangipuram and Giridhar, 2025), graph convolutional network (GCN) and Informer (Chen et al., 2025), integration of a Fourier kernel-based module into a deep residual network (DRN) combining artificial bee colony (Xu et al., 2026), integration of variational mode decomposition, Savitzky–Golay, and LSTM (Sun and Cai, 2022), residual neural network, and long short-term memory (Chen et al., 2023). Ensemble-based approaches, such as ensemble residuals for short-term load forecasting (Xu et al., 2020), have demonstrated better generalization and robustness in short-term load forecasting. However, the existing models often struggle to capture long-term dependencies and short-term fluctuations simultaneously.

Accurate modeling of long-range temporal dependencies in load forecasting remains complex, as load patterns depend on historical trends, periodic variations, and diverse exogenous factors. Existing studies struggle with long-range temporal dependencies and lack residual error correction. To address this challenge, a transformer model is employed to capture long-range temporal dependencies. The self-attention mechanism in the transformer architecture enables each time step to relate to other time steps to learn the historical trends and periodic variations effectively. This study proposes a two-stage attention-guided residual correction framework (TE-AG-GRU-RC) integrating a transformer encoder (TE) with an attention-guided GRU-based residual correction (AG-GRU-RC). The model has two stages; the framework enables the Stage 1 transformer encoder (TE) to capture long-term temporal dependencies, and Stage 2, with attention guidance and a fusion mechanism, explicitly corrects the baseline forecast errors and produces a refined and robust load forecast. The novelty of this work relies on its structured integration within a residual error modeling strategy, integrating global temporal representation learning with local sequential error refinement.

## 2 Related works

Although existing studies relied on traditional statistical and machine learning techniques, they faced challenges in handling non-linear temporal behavior, which reduced the performance of the model under complex circumstances and has encouraged researchers to transition toward neural networks. Even though forecasting accuracy is significantly improved with deep learning techniques, they face challenges in modeling long-term temporal dependencies, which can lead to a transition toward transformer-based models. Recent advancements in deep learning have introduced residual networks, transformer architectures, and attention mechanisms for handling time series data efficiently. Global temporal relationships can be effectively captured with transformers, and the majority of existing transformers rarely adapt to temporal complexity. The summary of recent forecasting approaches in STLF is discussed in Table 1.

**Table 1:** Comparative summary of recent short-term load forecasting models.

Author and year	Forecasting horizon	Dataset	Methodology	Metrics	Remarks
Sheng et al. (2023)	24-h horizon STLF	Independent System Operator-New England (ISO-NE) & Malaysia	Residual LSTM (DRN + LSTM) Dimension weighted unit	MAPE *R*^2^	• Avoids vanishing gradients and captures non-linear patterns effectively.
Yan et al. (2023)	1-h ahead STLF	Building 51G (residential users) at Arizona State University’s Tempe campus	FTTrans-E-BL and Pareto Optimization	RMSE MAE *R*^2^ Pinball loss	• Transformer and RNN architectures with a feature-time multi-headed attention to better capture temporal characteristics of multi-load. • Lack of interpretability.
Zheng et al. (2026)	Hourly forecasting horizon STLF	Single-story office building model in Tianjin, China	OB-TAM-LSTM	RMSE MAE MAPE *R*^2^	• Integration of occupant behavior and temporal attention enhances performance. • Lack of real-world data and meteorological features.
Han and Zeng (2025)	One day ahead STLF	University of California, Irvine (UCI) household electricity consumption dataset, Household Electricity consumption in a two-story Residence dataset from Houston, Texas, and Malaysia power company dataset	CEEMDAN Res-BiLSTM	RMSE MSE MAE MAPE NRMSE *R*^2^	• Model can extend the case study across diverse building. • Exploring advanced activation function can enhance generalization and minimize vanishing gradient risk.
Chen et al. (2021)	24-h ahead STLF	ISO-NE dataset, North-American Utility dataset	TDResNet Self-adaptive Dropout method	MAPE RMSE	• Handles vanishing and exploding gradients. • Future study can focus on self-adaptive adjustment using evolutionary algorithm. • High computational complexity
Laouafi et al. (2022)	24-h ahead STLF	Electricity demand data, Australian state of New South Wales	Calendar-based grouping–Median filtering –kNN–Pearson correlation–Ensemble forecasting–Gaussian error correction–support vector regression (CMKP-EG-SVR)	APE MAPE RMSE	• Integrates multiple preprocessing, feature selection, pattern extraction, ensemble forecasting, and error correction techniques under normal and anomalous load conditions. • Accuracy drops on extreme weather. • Focuses only on 24 h ahead forecast.
Huy et al. (2022)	24-h ahead STLF	Hanoi city electrical load (Hanoi Load Dispatch Center), weather data from Visual Crossing Corp.	TFT	MAE MAPE	• Weather and calendar features were utilized to improve the forecasting accuracy. • Lack of statistical significance testing • Lack of holidays and unexpected events.
Xiong (2024)	Hours to days STLF	UCI electrical power dataset	Residual-CNN + GRU Seq2Seq + Attention Mechanism + PCA	MSE, RMSE Smoothness score	• It enhances the load prediction performance by jointly exploiting temporal dependencies and feature interactions. • Lack of generalization.
Wang et al. (2025)	24-h ahead STLF	Multi-energy Integrated Energy System (IES) load dataset	Multi-scale memory enhanced transformer (MMEMformer)	MAE RMSE MAPE *R*^2^	• Combines memory-enhanced learning and cross-attention mechanisms to improve prediction stability • Interpretability of attention and memory retrieval remains to be explored.
Shi et al. (2017)	Few hours to days Household STLF	Smart Metering Electricity Customer Behavior Trials, Ireland	Pooling Deep RNN (LSTM-based)	RMSE NRMSE MAE	• Pooling strategy increases data diversity, reduces overfitting, and directly learns uncertainty. • Overfitting risk. • Lack of external features like weather.
Chen et al. (2018)	24-h ahead STLF	North-American utility dataset, ISO-NE dataset, Global Energy Forecasting Competition 2014 (GEFCom2014) dataset	Deep Residual Network (ResNet, ResNetPlus) + ensemble strategy	MAPE Out-of-range penalty (LR) Pinball loss Winkler score	• Model achieves generalization • Can explore deep neural networks with probabilistic STLF

### 2.1 Key contributions

The key contribution of the proposed model of this study for short-term load forecasting is summarized as follows.

- This study proposed a novel two-stage residual correction framework (TE-AG-GRU-RC) integrating a transformer encoder for modeling long-range temporal dependencies with a GRU-based residual corrector. This framework combines global context modeling with the local error dynamics to enhance the performance of short-term load forecasting.
- To ensure residual correction, attention guidance is introduced with a fusion mechanism combining the encoder projection of a contextual representation from the primary model with the GRU hidden states, enabling the correction path to integrate long-range temporal dependencies with the local sequential dynamics.
- Integrating a transformer encoder, attention guidance, fusion mechanism, and GRU-based residual correction enables the correction stage to focus on relevant temporal representations while refining the baseline forecast error.
- The proposed framework is validated on two geographically distinct datasets, demonstrating the generalizability across heterogeneous features and dynamic load patterns.

The remaining section of the paper is structured as follows. Section 3 describes the proposed methodology framework and key components. Section 4 defines the performance evaluation metrics used to evaluate the error. Section 5 discusses the forecasting results, comparative analysis, ablation studies, and discussions related to the model. Section 6 presents the conclusion and future scope.

## 3 Methodology

### 3.1 Three-layer outlier detection framework

Outliers were identified with a three-layer outlier detection framework that integrates a multi-layer strategy including hard rule-based detection, interquartile range (IQR)-based statistical detection, and Rolling *Z*-score detection technique.

#### 3.1.1 Layer 1: Hard rule-based detection

Initially, the physically impossible observations were removed with predefined domain constraints. Let *L_t_* denote the load demand at time *t*. If any observation satisfying the condition *L_t_* ≤ L_min_ (or) *L_t_* > *L*_max_ was identified as an outlier, where *L*_min_ is equal to 0 MW, and *L*_max_ is 3 * *P*_99,_ representing the 99th percentile of the load distribution.

#### 3.1.2 Layer 2: IQR-based detection

The remaining observations were investigated with the interquartile range (IQR) method. The quartiles are computed as shown in Equation 1, and the lower bound and upper bound are formalized as shown in Equations 2, 3.


$$\mathrm{IQR}=Q_{3}-Q_{1}$$ (1)

$$\text{Lower bound}\;(\mathrm{LB})=Q_{1}-1.5\;(\mathrm{IQR})$$ (2)

$$\text{Upper bound}\;(\mathrm{UB})=Q_{3}-1.5\;(\mathrm{IQR})$$ (3)

#### 3.1.3 Layer 3: Rolling *Z*-score detection

The rolling *z*-score was computed using a sliding window. Even though the IQR method detects global anomalies, it has challenges with local deviations caused by sudden demand changes. The rolling mean and standard deviation are represented as shown in Equations 4, 5:


$$\mu_{t}=\frac{1}{\omega }\sum_{i=1}^{\omega }l_{t}$$ (4)

$$\sigma_{t}=\sqrt{\frac{1}{\omega }\sum_{i=1}^{\omega }{\left(l_{i}-\mu_{t}\right)}^{2}}$$ (5)

where ω = sliding window, $\mu_{t}$ —mean, $l_{t}-\text{load}\;\mathrm{at}\;\text{time}\;t$ $\sigma_{t}$ —standard deviation.

The *z*-score is calculated as shown in Equation 6,


$$z_{t}=\frac{l_{t}-\mu_{t}}{\sigma_{t}}$$ (6)

The combined detection strategy obtains the final outlier by taking the union of three detection layers. After identifying the outliers, the corresponding observations were replaced with missing values and reconstructed with linear interpolation. This is followed by forward fill and backward fill operations when required to handle missing values, and linear interpolation is employed to preserve the continuity of the time series data and to avoid the discontinuities that cause degradation in the performance of the model.

### 3.2 Proposed two-stage residual correction (TE-AG-GRU-RC) framework

The proposed residual correction framework (TE-AG-GRU-RC) includes two stages as illustrated below.

Stage 1: Transformer encoder (TE)-based temporal representation learning and base forecasting.

Stage 2: Attention-guided GRU residual correction (AG-GRU-RC).

As depicted in Figure 1, the proposed framework follows a two-stage residual correction framework to improve forecasting accuracy by combining the transformer encoder (TE) baseline with a GRU-based residual learner. The proposed method begins with outlier detection and preprocessing, and the left and right sequences represent the baseline Transformer forecast and the residual correction pipeline, respectively. The final forecast is obtained by combining both predicted results. In Stage 1, the transformer encoder captures the long-term temporal dependencies through its multi-head self-attention mechanism. Feed-forward refinement produces a latent representation and yields the baseline forecast. This baseline forecast provides a stable reference for Stage 2 but does not fully capture the complex dynamics of load patterns. In Stage 2, the hidden state of *h*_gru_ is fused with the attention-guided encoder projection *h*_proj,_ and the attention guidance refers to the use of refined contextual information from the transformer encoder in Stage 2 while modeling the residual errors. This fusion enhances the GRU’s ability to learn the error dynamics systematically. This fused vector undergoes non-linear transformations, providing a dynamic latent state that is capable of capturing complex error signatures. Later, in the observation layer, the latent space is mapped into the forecast horizon to produce the residual corrections. During training, supervision is provided by minimizing the difference between the predicted and true residuals, enabling the GRU to learn the error distribution of the baseline forecast.

**Figure 1:**
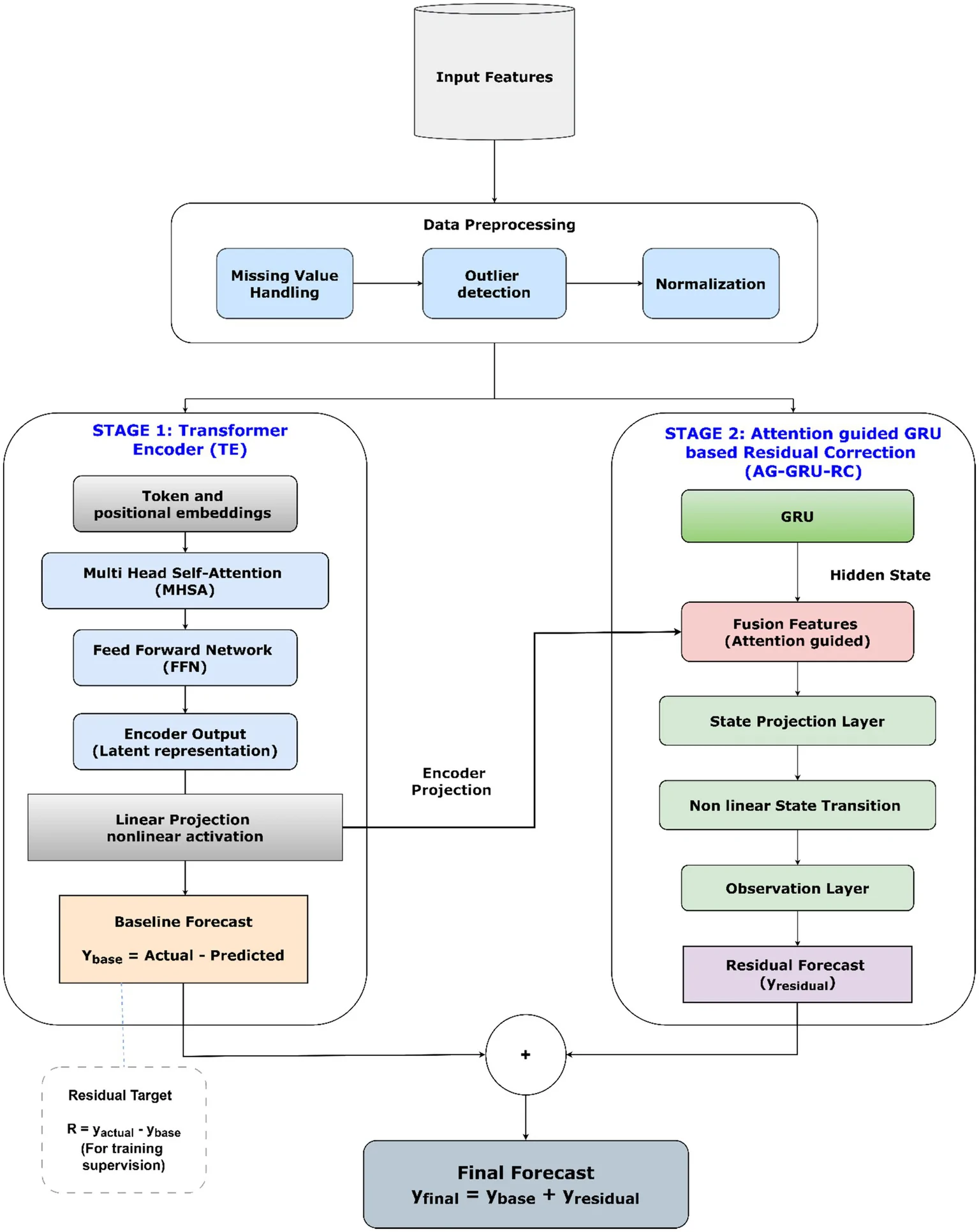
The proposed two-stage residual correction framework (TE-AG-GRU-RC) for short-term load forecasting.

#### 3.2.1 Stage 1: Transformer encoder (TE)-based temporal representation learning and base forecasting

The transformer encoder (TE) employs a self-attention mechanism to capture the high-dimensional temporal dynamics from historical energy demand, weather, and calendar features in the first stage. This stage generates both the initial baseline forecast and a latent representation that subsequently guides the residual correction process in Stage 2. The detailed pipeline of Stage 1 is outlined as follows:

Let the input sequence (*Z*) be represented as $Z=\{Z_{1},Z_{2},\ldots \ldots ,Z_{T}$ }, which is linearly projected into the model latent space and combined with the learned positional parameters to preserve temporal order, as represented in Equation 7,


$$E=W_{\text{embedding}}Z+\mathrm{PE}$$ (7)

where the input sequence is $Z\in R^{\text{Batch}\times \text{SeqXN}\_\text{features}}$ , the embedding matrix is $W_{\text{embedding}}\in R^{N\_\text{features}\times d\_\text{model}}$ , and positional encoding (PE) is $\in R^{B\times \mathrm{seq}\times d{\_}_{\text{model}}}$ .

After input and positional embedding, E is processed through stacked encoder layers.

Multi-head self-attention is applied at every layer, followed by a position-wise feed-forward network, with residual connections and normalization.

The embedded features are transformed into query (*Q*), key (*K*), and value (*V*) vectors and process the required information for one-head attention learning as shown in Equation 8.


$$\text{Attention}\;(Q,K,V)=\text{Softmax}\;(\frac{QK^{t}}{\sqrt{d_{k}}})V$$ (8)

where ‘*t*’ is the sequence length, $QK^{t}$ represents the measure of similarity between the positions, and $\sqrt{d_{k}}$ represents the head dimension and scaling factor for training stabilization.

The multi-head self-attention computes the attention scores between every pair of time steps in the sequence as formalized in Equation 9.


$$\text{MultiHeadSelfAttention}(I)=\text{Concat}({\text{head}}_{1},\ldots ,{\text{head}}_{h}).W_{\text{out}}$$ (9)

where *W*_out_ is the output projection matrix and subsequently, multiple heads compute attention in parallel subspaces, and the outputs are concatenated and linearly projected.

The output is passed to the feed-forward block, with residual connections and layer normalization enabling the model to capture long-range temporal dependencies and non-linear relationships across all time steps.

The embedded sequence is processed to produce the latent representation as represented in Equation 10 to capture the contextual dependencies across the input sequence completely.


H=Encoder(E) (10)

The encoder output is retained as an attention-aware latent representation containing global contextual information extracted by the self-attention mechanism. These latent features are subsequently used to guide residual learning in the second stage. The final encoder token ( $h_{\mathrm{enc}})$ is taken from the last time step of this sequence as given in Equation 11.


$$h_{\mathrm{enc}}=H[:,-1,:]\;$$ (11)

The final latent token encoder $\left(h_{\mathrm{enc}}\right)$ is linearly projected with non-linear activation as represented in Equation 12 to compress it into the GRU feature space in the second stage.


$$h_{\text{proj}}=\tanh\;(W_{\text{proj}}h_{\mathrm{enc}}+b_{\text{proj}})$$ (12)

This provides a bounded, smooth compression of encoder features, ensuring stable fusion with the GRU hidden state. Tanh introduces non-linearity and allows the projection to learn richer transformations.

The base forecast is then computed as mentioned in Equation 13.


$${\hat{y}}_{\text{base}}=W_{\text{base}}h_{\text{proj}}+b_{\text{base}}$$ (13)

The encoder is kept frozen during the second stage to preserve learned global representations. The frozen model is used in inference mode to produce fixed base forecasts and the corresponding final token encoder features ( $h_{\mathrm{enc}})\;$ for every window in the training, validation, and test sets. Once trained, Transformer parameters are frozen to preserve global attention patterns, and the residual correction stages focus only on error corrections. This ensures the GRU residual learner operates against a fixed baseline and improves interpretability and efficiency.

#### 3.2.2 Residual target calculation

The residuals are computed externally during training, defining the correction objective, and the residual target construction is represented as shown in Equation 14


$$\text{Residual}\;(R)=y-{\hat{y}}_{\text{base}}$$ (14)

These residual targets are computed as the difference between the ground truth and the Transformer baseline forecast. These residuals serve as supervisory signals for the GRU residual learner to compare and compute residual loss during training.

#### 3.2.3 Stage 2: Attention-guided GRU residual correction (AG-GRU-RC)

The input sequence (*Z*) is processed by a GRU network to produce a hidden representation encapsulating the local temporal dependencies, as shown in Equation 15, and subsequently fused with the encoder projection to guide the residual correction.


$$H_{\mathrm{gru}}=\mathrm{GRU}\;(X)$$ (15)

$$h_{\mathrm{gru}}=H_{\mathrm{gru}}[:,-1,:]$$ (16)

where [:, −1,:] in Equation 16 denotes take all batches, the last time step in the sequence, and take all hidden dimensions. The final hidden state summarizes short-term dependencies in the input sequence.

The encoder projection and the GRU hidden state are formalized in Equation 17 to combine global context with the local sequential dynamics.


$$h_{\text{fusion}}=\text{Fusion}[h_{\mathrm{gru}};h_{\text{proj}}]$$ (17)

where $h_{\text{proj}}$ is an attention-aware latent token fused with $h_{\mathrm{gru}}$ . This fusion ensures the residual learning is explicitly attention-guided, and the GRU residual learning integrates global contextual dependencies captured by self-attention with the local temporal dynamics.

As a residual state transformation, the fused representation undergoes successive non-linear transformations to form the dynamic latent state.

The fused representation $h_{\text{fusion}}$ is now passed to the state projection layer $S_{\text{proj}}$ , as mentioned in Equation 18, and it applies a linear transformation for the fusion of features into a standardized latent space.


$$S_{\text{proj}}=W_{\text{proj}}h_{\text{fusion}}+b_{\text{proj}}\;$$ (18)

This step compresses the fused features into a compact residual state space, enabling it to capture dynamic latent representations. The projected latent state undergoes a further non-linear transformation as represented in Equation 19.


$$S_{\text{trans}}=\text{ReLU}\;(W_{\text{trans}}S_{\text{proj}}+b_{\text{trans}})$$ (19)

This acts as a refinement step, stabilizing the residual representation and ensuring bounded corrections. The ReLU transformations allow the residual learner to capture nonlinear relationships and stabilize the latent space.

The refined latent space is passed through the linear observation layer and mapped into the forecast horizon to yield the residual forecast, as represented in Equation 20.


$${\hat{y}}_{\text{residual}}=w_{\mathrm{obs}}s_{\text{trans}}+b_{\mathrm{obs}}$$ (20)

This observation layer maps the transformed state into residual correction space, ensuring dimensional compatibility with the forecast target prediction length.

After residual correction, the final load forecast is produced by combining the predicted residual forecast and the baseline forecast (TE), as formalized in Equation 21.


$$y_{\text{final}}={\hat{y}}_{\text{base}}+{\hat{y}}_{\text{residual}}$$ (21)

The residual learner is trained by reducing the difference between the predicted residual forecast and the residual target calculated from the baseline forecast. The Huber loss function is employed to penalize the error to ensure robustness toward outliers and maintain sensitivity to small deviations. Finally, the estimated residual correction is added to the transformer baseline forecast to produce the final forecast.

### 3.3 Durbin–Watson (DW) statistical test

The residual learnability check is performed using the Durbin–Watson (DW) statistical test to investigate the residual autocorrelation, as described in Equation 22. This test determines whether the residuals of the primary forecasting model contain any systematic pattern that a secondary model can learn from to correct the errors. The DW statistical test detects the autocorrelation in the residuals, and the presence of autocorrelation means that the error at the current time step can be correlated with the past time steps. If the residuals were purely random noise, a corrector model would have nothing meaningful to learn.


$${\mathrm{DW}}_{\text{Statstics}}=\sum {\left(e_{t}-e_{t-1}\right)}^{2}/\sum {e_{t}}^{2}$$ (22)

where $e_{t}$ and $e_{t-1}$ are the residuals at time *t* and the previous time step (*t* − 1).

## 4 Performance evaluation metrics

To measure the prediction error and to evaluate the fitness of the model, the performance evaluation metrics are applied as represented in the Equations (23–27). Lower RMSE, MSE, MAE, and MAPE values are better, while $R^{2}$ with a higher value is better.

a) Mean Absolute Error (MAE)


$$=\frac{1}{m}\sum_{z=1}^{m}|{\text{Actual}}_{z}-{\text{Predicted}}_{z}|$$ (23)

b) Mean Squared Error (MSE)


$$=\frac{1}{m}\sum_{z=1}^{m}{\left({\text{Actual}}_{Z}-{\text{Predicted}}_{z}\right)}^{2}$$ (24)

c) Root Mean Squared Error (RMSE)


$$=\sqrt{\frac{1}{m}\sum_{z=1}^{m}{\left({\text{Actual}}_{z}-{\text{Predicted}}_{z}\right)}^{2}}\;\;$$ (25)

d) Mean Absolute Percentage Error (MAPE)


$$=\frac{100\%}{m}\sum_{z=1}^{m}|\frac{\left({\text{Actual}}_{z}-{\text{Predicted}}_{z}\right)}{{\text{Actual}}_{Z}}|$$ (26)

e) Coefficient of determination


$$\;(R^{2})=1-\frac{\sum_{z=1}^{m}{\left({\text{Actual}}_{z}-{\text{Predicted}}_{z}\right)}^{2}}{\sum_{z=1}^{m}{\left({\text{Actual}}_{z}-{\bar{\text{Actual}}}_{z}\right)}^{2}}$$ (27)

where ‘*m*’ represents the number of samples, ‘ ${\text{Actual}}_{z}$ ’ represents the actual value, ‘ ${\bar{\text{Actual}}}_{z}$ ’ represents the mean of actual values, and ‘ ${\text{Predicted}}_{z}$ ’ represents the predicted value.

## 5 Results and discussion

This section evaluates the proposed two-stage residual correction framework using two geographically distinct hourly load forecasting datasets: Tamil Nadu and Panama electricity load datasets. The performance evaluation of the model is examined with overall forecasting accuracy, ablation studies, multiple seed runs, and statistical tests.

### 5.1 Dataset description

The performance evaluation of this study includes two load datasets from two different geographical regions, including the Tamil Nadu electricity load dataset and the Panama electricity load dataset. The Tamil Nadu load dataset is the primary dataset used in the experimental evaluation, collected from the Indian Climate and Energy Dashboard (ICED), NITI Aayog, India (NITI Aayog & Vasudha Foundation, 2023). This dataset contains hourly demand for 7 years between January 2017 and April 2024, with 64,284 hourly observations of load in MW. These data are integrated with weather data collected from Open-Meteo historical weather API (Zippenfenig, 2023). The weather data are collected at district-level geographical coordinates of the Tamil Nadu region for every hourly time step. The weather features include apparent temperature, temperature, relative humidity, dew point, rain, weather code, precipitation, shortwave radiation, wind direction, and wind speed. All these weather features were combined with the load dataset. In addition, load demand (lag 1, 24, and 168), rolling demand features (24 and 168 h), and time-based features were also included in the dataset.

The Panama electricity load dataset is obtained from Kaggle (Aguilar Madrid and Antonio, 2021) as a secondary dataset to evaluate the effectiveness and generalizability of the model. The dataset contains 48,048 hourly observations for the period between 2015 and 2020. The dataset includes national electricity demand (MW) and meteorological features, such as air temperature, specific humidity, total liquid precipitation, and wind speed, recorded at three geographically distinct weather stations in Panama. In addition, calendar features, event-based features, and temporal features included demand lagged by 1, 24, and 168 h, and 24-h and 168-h rolling-mean demand averages were included.

### 5.2 Data preprocessing

Data preprocessing was performed on each dataset independently to ensure the quality and consistency of the data. The relevant features were extracted, and the raw data were prepared for model training. The missing value is handled with interpolation, especially for the lag features resulting from the feature engineering to support temporal dependencies. MinMaxScaler with the range of [0, 1] is used for normalization, and for outlier detection, a three-layer outlier detection approach is introduced to combine global and statistical decisions. Three-layer outlier detection integrates domain-specific constraints with statistical techniques to effectively identify the global and local anomalies present in the datasets, and this multi-layer technique minimizes false positives and protects the legitimate demand fluctuations. The hard-rule stage, considered as layer 1 of outlier detection, eliminates physically impossible measurements, and in layer 2, the IQR method identifies the statistically extreme observations across the entire dataset. The electrical load exhibits strong temporal dependencies and seasonal variations; hence, a rolling *Z*-score was incorporated in the third layer to detect localized anomalies that may not look like global outliers.

Initially, to ensure the robust assessment of the model performance, the training, validation, and test data split of the proposed model is in the ratio 70:15:15, as shown in Figure 2, for both datasets. The load distribution of the complete energy demand is shown in Figure 3.

**Figure 2:**
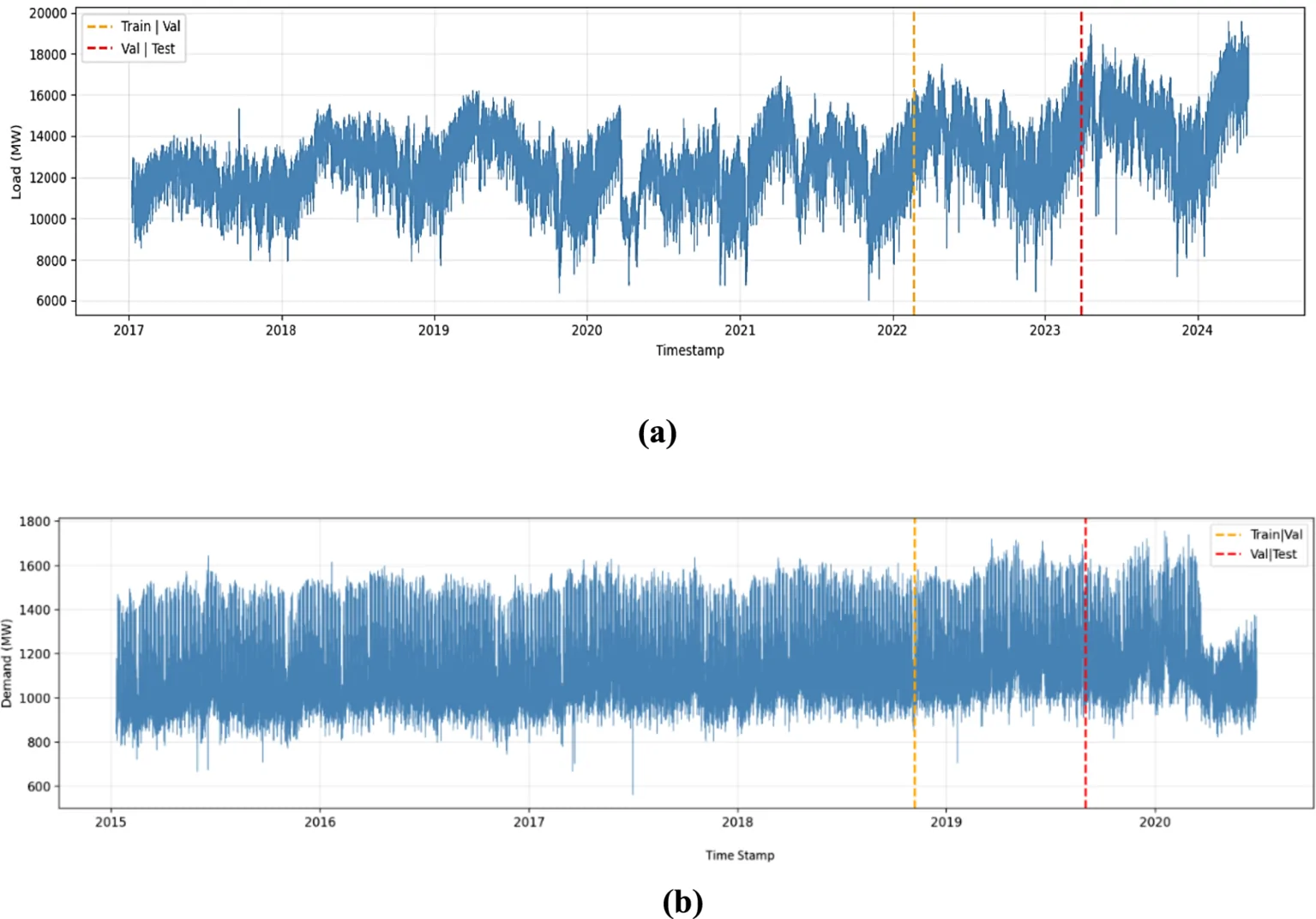
Data splitting strategy for training, validation, and testing sets on the **(a)** Tamil Nadu dataset **(b)** Panama dataset.

**Figure 3:**
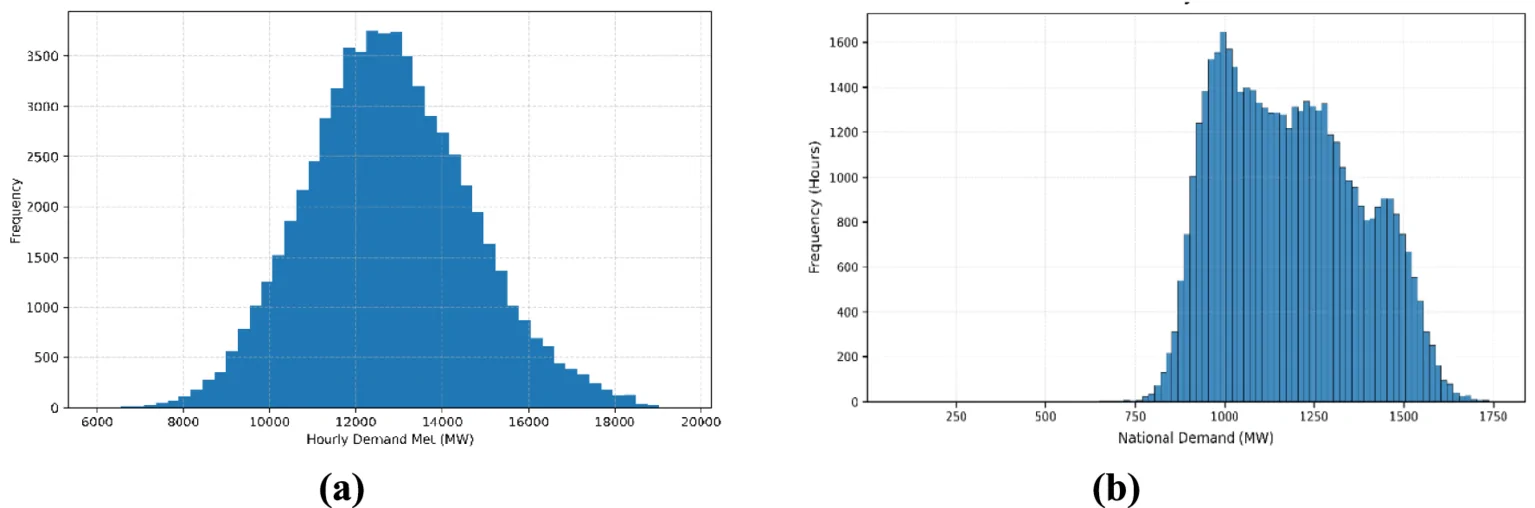
Histogram showing historical hourly load distribution **(a)** Tamil Nadu dataset and **(b)** Panama dataset.

In hard rules, flagged values are less than or equal to 0 MW or greater than three times the 99th percentile. The first layer detects no outliers within the valid range (0, 52,532.9) MW. The IQR fence detects 529 outliers, falling outside the range of 7,653.1 MW to 17,841.1 MW. Finally, the rolling *Z*-score method, using a 168-h window and a 3.5 threshold, additionally flags three outliers that were not identified by the previous layer. In total, 532 observations, equivalent to 0.83% of the dataset, were identified as outliers and treated with linear interpolation. The interpolation method supports smoothing out extreme values, retaining the overall trend and seasonality of the series. The outlier treatment was applied to all the lagged and rolling mean demand features derived from the target column by maintaining data consistency across relevant features. The multi-layer strategy employed for outlier detection ensures robust outlier handling and results in a clean dataset ready for model training and evaluation. The flagged outlier is shown in Figure 4a. This figure clearly shows the original hourly demand data (blue lines) over the entire time period. The red scattered points on the blue lines show that the specific data points were identified as flagged outliers (532 outliers). As shown in Figure 4b, the comparison between the original load series and the cleaned load series is depicted. The anomalies flagged as shown in Figure 4a have been smoothed out with linear interpolation. The orange line shows a consistent demand pattern free from extreme spikes and drops.

**Figure 4:**
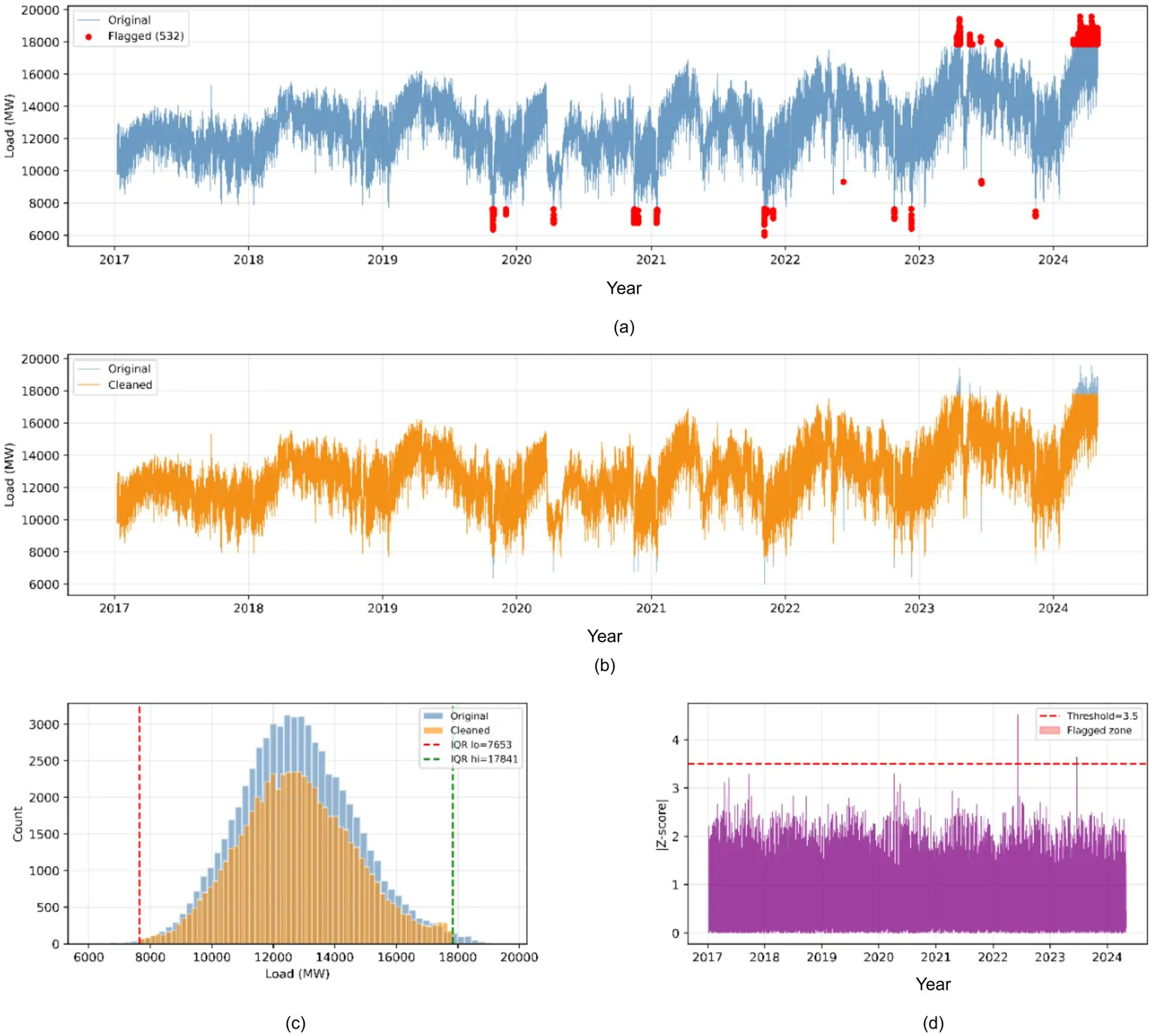
Three-layer outlier detection framework consisting of **(a)** flagged outliers on the hourly demand time period, **(b)** original Vs cleaned load series, **(c)** histogram distribution before and after outlier detection, and **(d)** rolling *Z*-score analysis for local anomaly identification.

The histogram describing the statistical distribution of hourly demand before and after outlier treatment is shown in Figure 4c. The red and green dashed lines denote the lower and upper boundaries of the IQR fence. Figure 4d shows the absolute rolling *z*-score of the hourly demand data over time. The red dashed line represents the *z*-score threshold of 3.5, and any points exceeding the threshold were identified as local anomalies. The figure indicates that the rolling score was calculated over a 168-h window for hourly demand. The benefit of using this three-layer outlier detection is described in Table 2. Similarly, for the Panama dataset, a three-level outlier assessment was performed on the national electricity demand (MW) variable using predefined validity limits, the interquartile range (IQR) criterion, and a 168-h rolling *Z*-score. Based on the observations, no values were identified outside the specified valid range, IQR, and *Z*-score threshold of 3.5. No modification is required, and the dataset was divided into a ratio of 70:15:15. The dataset includes 33,398 training data, 7,157 validation data, and 7,157 testing data, with the feature scaler on training data to maintain consistency and avoid leakage.

**Table 2:** Comparison of outlier detection approaches and their limitations with three-layer outlier detection.

Method	Limitations
Hard rules	Only physically impossible values are detected.
IQR	Global outliers are detected but ignore local temporal anomalies.
Rolling Z-score	Detects local anomalies but ignores global values.
Proposed three-layer outlier detection	Combines physical constraints and both local and global temporal behavior.

### 5.3 Experimental setup and evaluation

The hyperparameters employed in this model training are selected based on manual selection, validation performance, and computational considerations. The parameters described in Table 3 define the environment and fundamental settings of model training and evaluation. The model is executed in a GPU (CUDA) environment. The 168-h input window was selected to capture a complete weekly cycle of hourly observations, while the 24-h prediction horizon was defined according to the day-ahead forecasting objective. The remaining architectural and optimization settings were chosen to provide sufficient model capacity and stable training without unnecessary computational complexity.

**Table 3:** Model configuration and hyperparameter settings of the proposed (TE-AG-GRU-RC) model.

Parameters	Values	Description
Sequence length	168	Length of the historical input window (hours)—168 h, equal to 7 days, capturing a full weekly seasonal cycle in load demand.
Prediction length	24	Forecast horizon (hours): one full day ahead, standard for short-term load forecasting (STLF).
Loss function	Huber loss	Huber loss was used for residual correction
Optimizer	Adam	Adaptive moment estimation optimizer, chosen for stable convergence on non-stationary time series gradients without manual LR tuning per parameter.
Learning rate	le-3	Adam optimizer default
Epochs	100	Maximum training epochs per model, bounded by early stopping in practice.
Batch size	64	Number of sequence windows per gradient update, balancing GPU memory and gradient stability.
Early stopping patience	15	Number of epochs without validation-loss improvement before halting training, to prevent overfitting.
Dimensionality of model embeddings	128	Latent dimension
Attention heads	4	Number of self-attention heads in the transformer encoder.
Encoder layers	2	Depth of the transformer encoder stack.
Dimensionality of feed-forward network	256	Hidden dimension of the position-wise feed-forward sublayers within each encoder layer.
Dropout	0.2	Dropout rate applied across Transformer and GRU residual corrector modules to reduce overfitting.
Latent dimension	64	Latent state dimension of the GRU residual corrector internal state transition representation.
Number of layers	2	Depth of the GRU stack layers in the residual correction module.

To improve reproducibility, the detailed configurations and hyperparameters of all baseline models are included. Specifically, the RNN, LSTM, and GRU models were configured with a hidden size of 128, two recurrent layers, and a dropout rate of 0.2, with a linear output layer mapping the final hidden state to the 24-h forecasting horizon. The TFT model was configured with an LSTM-based encoder, a model dimension of 128, one encoder layer, four attention heads, and a dropout rate of 0.2, followed by a linear output layer. All baseline models used an input sequence length of 168 time steps and a prediction horizon of 24 time steps. The models were trained under a common training protocol using the Adam optimizer with a learning rate of 1e−3, a batch size of 64, a maximum of 100 epochs, and early stopping with a patience of 15. These details have been added to the revised manuscript to provide a clear and reproducible basis for the comparative evaluation. We reimplemented Residual LSTM Plus (Sheng et al., 2023) as a baseline model on our datasets with identical data preprocessing and evaluation settings, with Huber loss, early stopping, and learning rate scheduling to suit the datasets. This model combines residual connections with the stacked LSTM and dimension weighted unit (DWU) to capture temporal and feature dependencies.

#### 5.3.1 Performance comparison results on the Tamil Nadu and Panama datasets

The comparative analysis of the performance of the proposed model against other baseline models for the Tamil Nadu dataset and Panama dataset is described in Tables 4, 5. The proposed model achieves the lowest error across all other models based on the error metrics, such as MAE, MSE, RMSE, MAPE, and *R*^2^. The result obtained demonstrates that the proposed (TE-AG-GRU-RC) outperforms other baseline models used for comparison. The findings show that the proposed model enhances forecasting robustness by combining global temporal information with a GRU residual corrector to refine the model. The proposed framework combines the strengths of attention mechanisms for global pattern recognition and recurrent networks for sequential modeling, while the residual learning approach ensures effective error correction. Similarly, for the Panama dataset, all the models are evaluated under an identical input configuration, including a 168-h (7-day) historical lookback window used to forecast the subsequent 24-h (1-day) demand horizon, enabling direct architectural comparison across datasets with differing geographic, climatic, and demand-pattern characteristics. The proposed TE-AG-GRU-RC achieves the lowest error across all baseline models, as shown in Table 5.

**Table 4:** Performance comparison of the proposed TE-AG-GRU-RC against baseline models on the Tamil Nadu dataset.

Model	MAE	MSE	RMSE	MAPE	*R* ^2^
TE-AG-GRU-RC	0.0297	0.0014	0.0376	3.58	0.879
Residual LSTM Plus (Sheng et al.)	0.0588	0.0058	0.0762	3.68	0.866
TFT	0.0699	0.0078	0.0888	4.30	0.818
GRU	0.0630	0.0065	0.0806	4.01	0.850
LSTM	0.0716	0.0084	0.0918	4.39	0.805
RNN	0.0658	0.0071	0.0844	4.08	0.836

**Table 5:** Performance comparison of the proposed model TE-AG-GRU-RC against baseline models on the Panama dataset.

Model	MAE	MSE	RMSE	MAPE	*R* ^2^
TE-AG-GRU-RC	0.0334	0.0023	0.0482	4.99	0.790
Residual LSTM Plus (Sheng et al.)	0.0575	0.0066	0.0818	5.35	0.769
TFT	0.0553	0.0061	0.0779	5.11	0.791
GRU	0.0594	0.0066	0.0815	5.52	0.770
LSTM	0.0624	0.0073	0.0853	5.76	0.749
RNN	0.0584	0.0066	0.0812	5.44	0.772

The proposed TE-AG-GRU-RC model outperforms baseline models across both datasets with an MAE of 0.0297, an MSE of 0.0014, an RMSE of 0.0376, an MAPE of 3.58, and an *R*^2^ of 0.879 for the Tamil Nadu dataset, and an MAE of 0.0334, an MSE of 0.0023, an RMSE of 0.0482, an MAPE of 4.99, and an *R*^2^ of 0.790 on the Panama dataset.

### 5.4 Ablation studies on the Tamil Nadu and Panama load datasets

To examine the contribution of each component, an ablation study was conducted for the two-stage forecasting framework. In particular, the ablation analysis investigates whether the performance improvement of the proposed framework arises solely from the base forecasting architecture or from the complementary interaction between the base forecast and the residual correction stage. By progressively comparing the GRU, transformer Encoder, transformer–GRU residual correction model (TE-GRU-RC) without attention guidance, and the full proposed model (TE-AG-GRU-RC), the analysis quantifies the effect of each architectural enhancement through changes in the reported evaluation metrics. The results demonstrate the extent to which residual learning provides additional predictive information by modeling the remaining discrepancy not captured by the initial forecast, while the comparison with the complete model further evaluates the contribution of the attention-guided residual mechanism. Therefore, the ablation findings should be interpreted based on consistent improvements across the complete test set rather than on individual forecast examples, providing evidence that the final prediction accuracy results from the complementary contributions of the base forecasting and residual correction components (Tables 6, 7).

**Table 6:** Ablation study results evaluating the contribution of individual architectural components to forecasting performance on the Tamil Nadu dataset.

Model	MAE	MSE	RMSE	MAPE	*R* ^2^
GRU	0.0630	0.0065	0.0806	4.01	0.850
Transformer encoder	0.0612	0.0059	0.0771	3.87	0.863
TE-GRU-RC	0.0321	0.0016	0.0404	3.89	0.860
TE-AG-GRU-RC	0.0297	0.0014	0.0376	3.58	0.879

**Table 7:** Ablation study results evaluating the contribution of individual architectural components to forecasting performance on the Panama dataset.

Model	MAE	MSE	RMSE	MAPE	*R* ^2^
GRU	0.0594	0.0066	0.0815	5.52	0.770
Transformer encoder	0.0594	0.0068	0.0827	5.48	0.764
TE-GRU-RC	0.0338	0.0024	0.0495	5.06	0.779
TE-AG-GRU-RC	0.0334	0.0023	0.0482	4.99	0.790

### 5.5 Statistical validation of model performance

#### 5.5.1 Multi-seed performance stability analysis

To examine the robustness of the proposed framework to stochastic training effects, repeated independent training runs were conducted using different random seeds for both the Tamil Nadu and Panama datasets. The chronological training, validation, test partitions, model architecture, and all other experimental settings were kept unchanged across runs, while the random seed was varied to account for differences arising from stochastic parameter initialization and mini-batch optimization. Across five independent runs, the performance of the model is reported as the mean, standard deviation, and coefficient of variation (CV) of evaluation metrics MAE, MSE, RMSE, MAPE, and *R*^2^. The evaluation results show the consistency of the observed performance and preserve the temporal structure of the forecasting problem (Table 8).

**Table 8:** Seed-independent runs summary of the proposed TE-AG-GRU-RC framework on both datasets.

Seed	Tamil Nadu					Panama				
	MAE	MSE	RMSE	MAPE	*R* ^2^	MAE	MSE	RMSE	MAPE	*R* ^2^
42	0.0356	0.0021	0.0458	4.31	0.821	0.0359	0.0026	0.0512	5.39	0.763
123	0.0365	0.0022	0.0466	4.37	0.814	0.0345	0.0025	0.0502	5.15	0.772
2024	0.0337	0.0018	0.0427	4.06	0.844	0.0339	0.0025	0.0497	5.08	0.777
7	0.0338	0.0019	0.0432	4.05	0.840	0.0343	0.0025	0.0496	5.16	0.778
999	0.0349	0.0019	0.0445	4.23	0.831	0.0352	0.0026	0.0511	5.29	0.764
Mean ± Std	0.0349 ± 0.0012	0.0019 ± 0.0001	0.0446 ± 0.0016	4.20 ± 0.1458	0.830 ± 0.012	0.0347 ± 0.0007	0.0025 ± 0.0001	0.0504 ± 0.0007	5.22 ± 0.1259	0.771 ± 0.006
CV (%)	3.45	7.36	3.68	3.47	1.51	2.24	2.99	1.49	2.41	0.89

The model was trained with five different seeds (including 42, 123, 2024, 7, and 999) for both datasets to ensure robustness toward random initialization. For Tamil Nadu, the mean MAE was 0.0349 ± 0.0012, RMSE was 0.0446 ± 0.0016, MAPE was 4.21% ± 0.15%, and *R*^2^ was 0.830 ± 0.012 (scaled [0, 1]), with coefficients of variation (CVs) between 1.51 and 7.36%. For Panama, the mean MAE was 0.0348 ± 0.0008, RMSE was 0.0504 ± 0.0008, MAPE was 5.22% ± 0.13%, and *R*^2^ was 0.771 ± 0.007, with CVs between 0.89 and 2.99%, consistently lower than Tamil Nadu’s. Comparing Tamil Nadu and Panama in terms of *R*^2^, the Tamil Nadu dataset shows higher mean accuracy than the Panama dataset (0.830 vs. 0.771). Despite this, the Panama dataset shows lower seed variability across every metric, indicating that the accuracy is not accompanied by reduced training stability. Both the dataset results low CVs, including <7.4% for Tamil Nadu dataset and <3.0% for the Panama dataset, show that the reported gains are architectural and not due to favorable initialization.

#### 5.5.2 Confidence interval (CI) estimation

The model was trained and evaluated over five independent runs using different random seeds to evaluate the robustness of the proposed framework related to stochastic training variability. Table 9 shows the mean performance and its corresponding 95% confidence interval calculation using the t-distribution. The proposed model on the Tamil Nadu dataset results in a mean MAE of 0.0349 and a 95% CI of [0.0334, 0.0364]. Similarly, MSE was 0.0019 with a CI of [0.0018, 0.0021], a mean RMSE of 0.0446 with a CI of [0.0425, 0.0466], and a mean *R*^2^ of 0.8302 with a CI of [0.8147, 0.8457]. The relatively narrow confidence intervals show that the forecasting performance is consistent across five independent seed runs. Furthermore, the maximum half-widths of the CI were ±0.0015 for MAE, ±0.00018 for MSE, ±0.0020 for RMSE, and ±0.0155 for *R*^2^. The findings indicate that the reported performance is not dependent on a particular random seed. The result shows that the forecasting performance of the proposed model demonstrates reasonable stability under repeated stochastic training conditions.

**Table 9:** Confidence interval estimation for the proposed TE-AG-GRU-RC framework on both datasets.

Metrics	Tamil Nadu		Panama	
	Mean	95% CI	Mean	95% CI
MAE	0.0349	[0.0334, 0.0364]	0.0348	[0.0338, 0.0357]
MSE	0.0019	[0.0018, 0.0021]	0.0025	[0.0024, 0.0026]
RMSE	0.0446	[0.0425, 0.0466]	0.0504	[0.0494, 0.0513]
MAPE	4.2054	[4.02, 4.39]	5.2182	[5.06, 5.37]
*R* ^2^	0.8302	[0.8147, 0.8457]	0.7711	[0.7626, 0.7796]

On the Panama dataset, the proposed TE-AG-GRU-RC model attains a mean MAE of 0.0348 with CI [0.0338, 0.0357], MSE of 0.0025 with CI [0.0024, 0.0026], RMSE of 0.0504 with CI [0.0494, 0.0513], and *R*^2^ of 0.771 with CI [0.763, 0.780]. The confidence intervals across all the metrics show that the performance is statistically stable and reproducible across multiple seed runs. This shows robustness to random initialization and reinforces the reliability of the improvements reported.

#### 5.5.3 Computational efficiency and inference performance

In addition to forecasting accuracy, the computational requirements of the evaluated models were examined in terms of training parameters, training time, and inference latency per sample. This analysis provides complementary evidence regarding the practical efficiency of the forecasting models. The forecasting architecture remains the same for both datasets, while the input-dependent layers are configured according to the respective input feature dimensionality. Hence, the input feature dimensionality varies across the two datasets, leading to small variations in trainable parameter count (Table 10).

**Table 10:** Comparison of training parameters, training time, and inference latency of the evaluated forecasting models.

Model	Tamil Nadu			Panama		
	Trainable parameters	Training time (min)	Inference latency (ms/sample)	Trainable parameters	Training time (min)	Inference latency (ms/sample)
RNN	55,192	1.2	0.018 ± 0.003	55,832	2.21	0.029 ± 0.004
LSTM	211,480	2.0	0.051 ± 0.006	214,040	5.25	0.055 ± 0.013
GRU	159,384	2.3	0.034 ± 0.002	161,304	2.8	0.037 ± 0.003
TFT	2,234,647	18.7	0.514 ± 0.019	2,678,192	29.35	0.609 ± 0.026
Residual LSTMPlus (Sheng et al.)	5,117,360	35.3	0.814 ± 0.117	5,333,680	29.3	0.788 ± 0.109
TE-AG-GRU-RC	487,088	11.1	0.123 ± 0.007	489,648	1.7	0.126 ± 0.009

#### 5.5.4 Statistical significance testing

The Diebold–Mariano test was performed as a statistical significance test on both datasets to examine the difference in forecasting accuracy between the proposed model and other baseline models used for comparison. The results obtained from the evaluation metrics MAE, MSE, RMSE, MAPE, and *R*^2^ show the magnitude of forecasting errors; however, the statistical test is required to validate the observed performance difference. The DM test addresses this limitation by comparing the loss differentials derived from the forecast errors of two competing models over the same forecast evaluation period. Accordingly, the test was used to assess whether the proposed model provides a statistically significant improvement in forecasting performance relative to the baseline and comparative models (Table 11).

**Table 11:** Diebold–Mariano test results comparing forecast accuracy between the proposed TE-AG-GRU-RC framework and baseline models on both datasets.

Baseline Models	Tamil Nadu			Panama		
	DM statistic	*p*-value	Significant (*α* = 0.001)	DM statistic	*p*-value	Significant (*α* = 0.001)
RNN	33.36	0.000000	Statistically significant at *α* = 0.001 and *p*-value <0.001	15.51	0.000000	Statistically significant at *α* = 0.001 and *p*-value <0.001
LSTM	36.38	0.000000		17.18	0.000000	
GRU	20.26	0.000000		14.43	0.000000	
TFT	33.49	0.000000		11.71	0.000000	
Residual LSTMPlus (Sheng et al.)	10.35	0.000000		11.09	0.000000	

#### 5.5.5 Residual autocorrelation analysis

The Durbin–Watson (DW) statistical test was performed on the Tamil Nadu dataset, and the obtained result was 0.2266, indicating a strong positive autocorrelation. The autocorrelation values are significant for the observed lag 1 (0.8694) and lag 24 (0.8768), showing that short-term and daily recurring patterns are present within the residual sequence, as shown in Figures 5a,b. The Durbin–Watson (DW) statistic ranges from 0 to 4. The DW statistical test sets the condition as follows: if DW is equal to 2, it represents no correlation. If DW is less than 2, it indicates a positive correlation. Similarly, when DW is greater than 2, it indicates a negative correlation. For the Tamil Nadu dataset, the DW score attained is 0.2266, which is close to zero and indicates a strong positive autocorrelation. This shows that the residuals are not random noise and still contain the predictable temporal pattern information needed to be learned in the second stage. These results show that the forecasting errors are not random and can be used in the second stage for a residual learning framework to improve the performance of the model.

**Figure 5:**
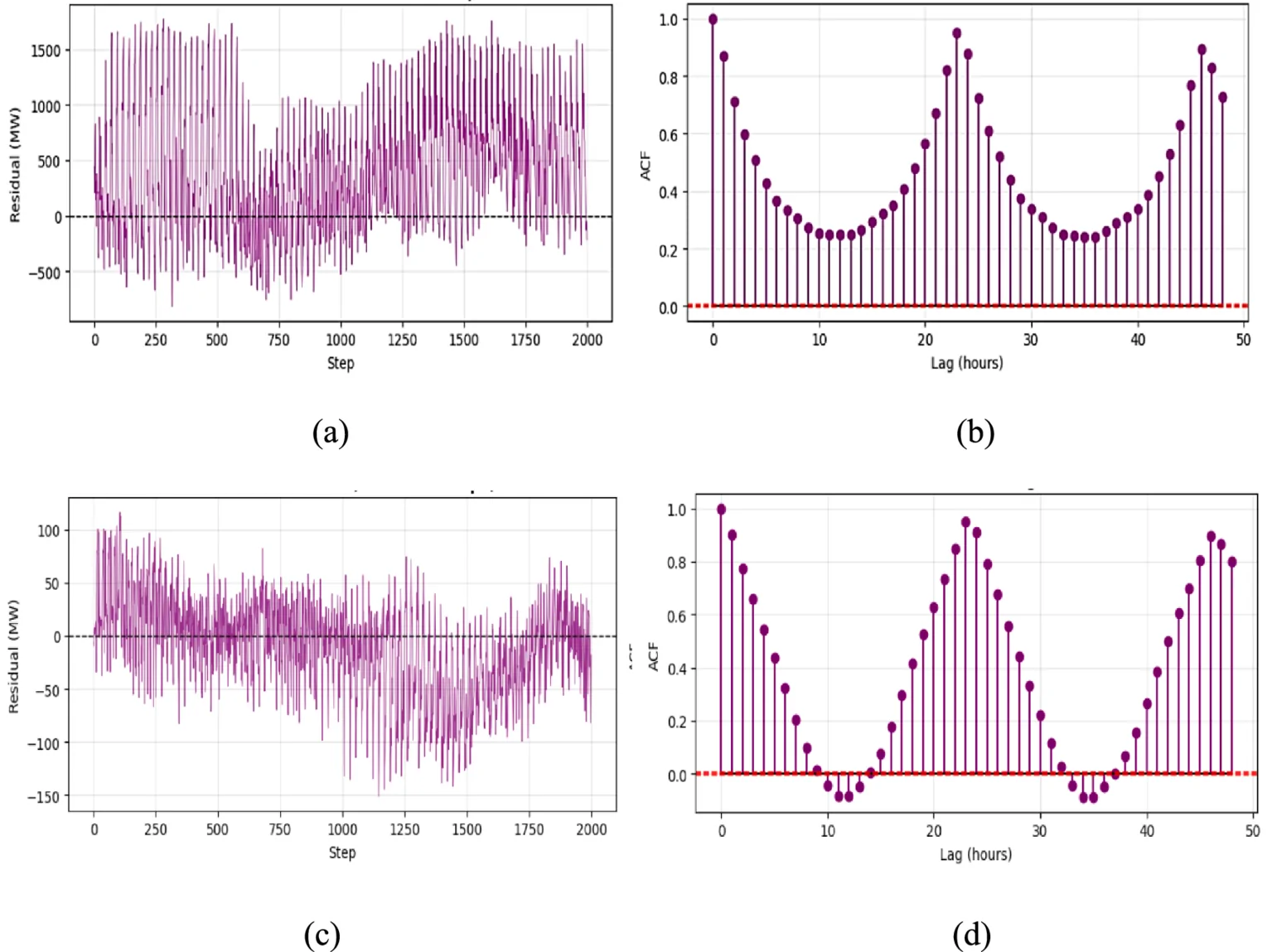
Residual learnability check with Durbin–Watson statistical test, including **(a)** Tamil Nadu–residual time series plot, **(b)** Tamil Nadu–ACF of residuals for lags (0–48), **(c)** Panama–residual time series plot, and **(d)** Panama–ACF of residuals for lags (0–48).

Similarly, the Durbin–Watson statistical test was performed on the Panama dataset, and the DW statistic score attained was 0.1924, with the autocorrelation at lag 1 (ACF = 0.9020) and lag 24 (ACF = 0.9106), indicating that the residual information is retained. This persistence shows that the remaining forecasting error is structured systematically rather than randomly and provides a strong reason for applying a residual correction framework to refine the transformer encoder baseline forecast. The residual time series plot and autocorrelation of residuals are depicted in Figures 5c,d.

Additionally, we have examined all test data on the Tamil Nadu dataset to determine the residual forecasting errors under dynamic load conditions. The correlation between the hourly load variation and absolute error in Stage 1 for 1-h was 0.2717, indicating that larger load variations tend to be associated with increasing Stage 1 errors. For instance, a representative forecast window on 1 May 2023 is considered for analysis. At 05.00, the actual load was 12,309.17 MW, and after the Stage 1 prediction, it the baseline forecast was 12,743.56 MW, and the absolute error was 434.39 MW. In Stage 2, the error was reduced to 411 MW. This illustrates how residual correction improves forecasts under dynamic load conditions.

Figures 6a,b illustrate the comparison of actual versus predicted 24-h-ahead demand curves for the Tamil Nadu and Panama datasets. The proposed (TE-AG-GRU-RC) model load curve was closer to the actual load curve than other baseline models. The closer alignment between the actual and predicted load curve indicates that the proposed model outperforms other comparison models and enhances the ability to capture complex temporal patterns in both datasets.

**Figure 6:**
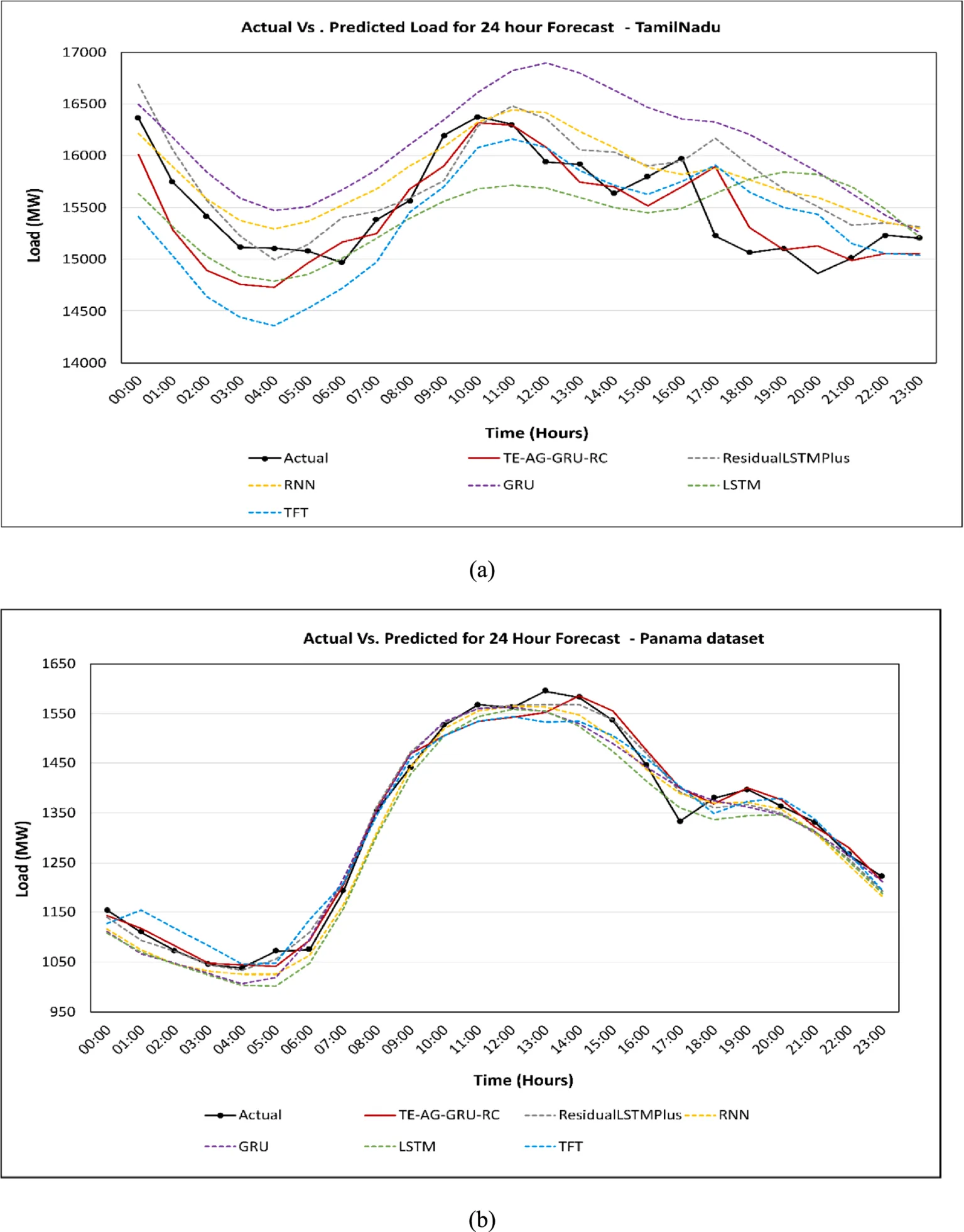
Comparison of 24-h actual versus predicted load curve: **(a)** Tamil Nadu dataset **(b)** Panama dataset.

## 6 Conclusion and future enhancement

Accurate short-term load forecasting plays a major role in effective power planning and operations, scheduling, and energy management under dynamic load patterns. This study proposed a novel two-stage residual correction framework (TE-AG-GRU-RC) for 24-h-ahead short-term load forecasting. The integration of a transformer encoder base with the attention-guided GRU residual learner effectively corrected the systematic errors and outperformed other baseline models. The proposed model addresses the challenge of simultaneously capturing the complex temporal dynamics and reducing the forecasting error in short-term load forecasting. In Stage 1, the transformer encoder (TE) learns the temporal dynamics in the load pattern, and this information is incorporated in Stage 2 through an attention-guided fusion mechanism. The Stage 2 attention-guided GRU (AG-GRU-RC)-based residual correction learns the residual to model the remaining error of the baseline forecast. The residual target computes the difference between the actual and baseline prediction, providing supervision for residual learning. The fused representation is transformed through a projection layer and transition to forecast the residual. The final load forecast is predicted by combining the baseline with the predicted residual.

The evaluation result on two dataset shows the proposed model outperformed across other baseline models RNN, GRU, LSTM, TFT, and ResidualLSTMPlus achieving an MAE of 0.0297, an MSE of 0.0014, an RMSE of 0.0376, an MAPE of 3.58, and an *R*^2^ of 0.879 on the Tamil Nadu dataset and achieving an MAE of 0.0334, an MSE of 0.0023, an RMSE of 0.0482, an MAPE of 4.99, and an *R*^2^ of 0.790 on the Panama dataset. The proposed model achieved lower MAE, MSE, MAPE, and RMSE values and higher *R*^2^ scores, supported by multiple seed runs producing a narrow confidence interval, confirming its robustness and reproducibility. The consistent improvement in both the geographically and climatically distinct datasets shows the generalizability of the model and supports sustainability by enabling more efficient energy management and reducing waste in the power systems. The ablation studies provide evidence on the contribution of components of the proposed model to the overall performance of the forecasting. The consistent results show that the model can accommodate different load patterns of different geographical regions. Even though the input layers are configured based on the input features and their dimensionality, forecasting accuracy is achieved.

Future studies can extend to longer forecasting horizons and incorporate probabilistic and peak-sensitive analysis to provide uncertainty estimation and enhance decision-making in energy systems by developing a visualization tool for attention weights and residual corrections to enhance explainability. Incorporating adaptive hyperparameter tuning can further improve the robustness of the model under dynamic conditions.

## Data Availability

The raw data supporting the conclusions of this article will be made available by the authors, without undue reservation.

## Glossary

Acronyms
ACF: Autocorrelation function
ANN: Artificial neural network
APE: Absolute percentage error
API: Application programming interface
ARIMA: Autoregressive integrated moving average
CEEMDAN: Complete ensemble empirical mode decomposition with adaptive noise
CNN: Convolution neural network
CV: Computer vision
DRN: Deep residual network
DWU: Dimension weighted unit
DW: Durbin–Watson
FTTrans-E-BL: Feature-time transformer encoder bidirectional long short-term memory
LightGBM: Light gradient boosting memory
MW: Megawatt
NRMSE: Normalized root mean square error
OB-TAM-LSTM: Occupant behavior–temporal attention mechanism–long short-term memory
PCA: Principal component analysis
Res-BiLSTM: Recurrent bidirectional long short-term memory
SARIMA: Seasonal autoregressive integrated moving average
TCN: Temporal convolutional network
TDResNet: Trilinear deep residual network
TE-AG-GRU-RC: Transformer encoder and attention-guided GRU-based residual corrector
Transformer-E-Bi-LSTM: Transformer-based encoder-bidirectional long short-term memory
WTLCC: Windowed time-lagged cross-correlation
XGBoost: eXtreme gradient boosting
Symbols
*Q*: Query matrix
*K*: Key matrix
*V*: Value matrix
$d_{k}$: Dimension of the key vector
*W*_out_: Output projection matrix
$h_{\mathrm{enc}}$: Transformer encoder hidden state
$h_{\text{proj}}$: Linear projection of encoder hidden state
$W_{\text{proj}}$: Projection weight matrix
$b_{\text{proj}}$: Bias vector for projection
${\hat{y}}_{\text{base}}$: Baseline forecast from projected encoder output
$W_{\text{base}}$: Weight matrix for baseline forecast from encoder output
$b_{\text{base}}$: Bias tern for baseline forecast projection
$H_{\mathrm{gru}}$: Hidde state matrix for GRU
$h_{\mathrm{gru}}$: Final hidden state vector for GRU
$h_{\text{fusion}}$: Fusion vector
$S_{\text{proj}}$: Projected residual state vector
$S_{\text{trans}}$: Transformer residual state vector
$W_{\text{trans}}$: Weight matrix for residual state transformation
$b_{\text{trans}}$: Bias term for residual state transformation
${\hat{y}}_{\text{residual}}$: Residual forecast correction
$w_{\mathrm{obs}}$: Output weight matrix
$y_{\text{final}}$: Final corrected forecast combining baseline and residual correction
